# Discordant congenital heart defects in monochorionic twins: Risk factors and proposed pathophysiology

**DOI:** 10.1371/journal.pone.0251160

**Published:** 2021-05-06

**Authors:** Helia Imany-Shakibai, Ophelia Yin, Matthew R. Russell, Mark Sklansky, Gary Satou, Yalda Afshar

**Affiliations:** 1 David Geffen School of Medicine, UCLA, Los Angeles, California, United States of America; 2 Division of Maternal Fetal Medicine, Department of Obstetrics and Gynecology, UCLA, Los Angeles, California, United States of America; 3 Department of Pediatrics, Kaiser Permanente Southern California, Los Angeles, California, United States of America; 4 Division of Pediatric Cardiology, UCLA Mattel Children’s Hospital, Los Angeles, California, United States of America; Baylor College of Medicine, UNITED STATES

## Abstract

A six-fold increase in congenital heart defects (CHD) exists among monochorionic (MC) twins compared to singleton or dichorionic twin pregnancies. Though MC twins share an identical genotype, discordant phenotypes related to CHD and other malformations have been described, with reported rates of concordance for various congenital anomalies at less than 20%. Our objective was to characterize the frequency and spectrum of CHD in a contemporary cohort of MC twins, coupled with genetic and clinical variables to provide insight into risk factors and pathophysiology of discordant CHD in MC twins. Retrospective analysis of all twins receiving prenatal fetal echocardiography at a single institution from January 2010 –March 2020 (N = 163) yielded 23 MC twin pairs (46 neonates) with CHD (n = 5 concordant CHD, n = 18 discordant CHD). The most common lesions were septal defects (60% and 45.5% in concordant and discordant cohorts, respectively) and right heart lesions (40% and 18.2% in concordant and discordant cohorts, respectively). Diagnostic genetic testing was abnormal for 20% of the concordant and 5.6% of the discordant pairs, with no difference in rate of abnormal genetic results between the groups (p = 0.395). No significant association was found between clinical risk factors and development of discordant CHD (p>0.05). This data demonstrates the possibility of environmental and epigenetic influences versus genotypic factors in the development of discordant CHD in monochorionic twins.

## Introduction

Congenital heart defects (CHD) are the most prevalent group of congenital anomalies, affecting approximately 0.9% of all singleton births [1, 2]. In monochorionic (MC) twins, the prevalence of CHD is six times higher, affecting 59 per 1000 live births [1].

Despite sharing an identical genotype, MC twins can develop discordant phenotypes for congenital malformations including CHD. Studies have not found strong genetic influences on discordant CHD in MC twins, with attributable genetic causes epigenetic in origin as opposed to differences in germline mutations or different phenotypic expression of the same genotype [3, 4]. It has been hypothesized that environmental influences such as teratogens can interfere with epigenetic processes leading to differential gene expression and discordant CHD [5].

In addition to epigenetics, local placental influences have also been identified as significant factors contributing to discordant CHD [5]. A 2011 study reported that 41% of all studied cases of discordant CHD resulted from placenta-related pathophysiologic mechanisms [5]. It has been hypothesized that placental inter-twin vascular connections, cord insertion sites, and relative placental-share contribute to imbalance of blood flow leading to relative hypoperfusion of one twin [5].

A major risk factor unique to MC twin population is the development of twin-to-twin transfusion syndrome (TTTS), an anomaly of placental vascular anastomoses causing an imbalance of blood flow from the donor twin to the recipient twin. The rate of CHD rises to 9.3% in MC pregnancies complicated by TTTS [6]. In the setting of TTTS, the imbalance of blood flow and increased aortic velocity in the recipient twin leads to increased risk of structural CHD in the recipient twin compared to the donor twin [5, 7]. Although several factors have been linked to the development of discordant CHD in MC twins, there is currently an incomplete understanding of the pathophysiology and associated risk factors of this diagnosis [3, 5].

The importance of early identification for CHD has been demonstrated by previous studies [8–11]. Although many cases critical CHD diagnoses occur either prenatally or subsequently through newborn screening, one in five cases of critical CHD are not diagnosed until after the fourth week of life [10]. Despite advances in fetal echocardiography, up to 60% of CHD diagnoses are diagnosed postnatally [12, 13]. Late diagnosis of critical CHD is associated with increased risk of morbidity and mortality, increased hospital length of stay, and 35% higher inpatient costs during infancy [8, 9, 11]. Therefore, understanding the pathophysiology and risk factors associated with discordant CHD in MC twins is critical to allow for early evaluation of an at-risk fetus. Early detection would work to reduce delivery and postnatal complications and aid in prevention through identification of modifiable risk factors.

This study examines fetal and maternal variables comparing MC twin pairs discordant versus concordant for CHD. We aim to describe the spectrum of lesions in this population and to elucidate risk factors and pathophysiology of discordant CHD in a modern cohort of MC twins. The monochorionic twin pair serves as an ideal model to evaluate environmentally triggered errors in cardiac development that contribute to CHD, both in the multifetal pregnancy as well as singleton pregnancies.

## Methods

This retrospective cohort study utilized all prenatal screening echocardiograms conducted at the University of California, Los Angeles (UCLA) from January 2010 –March 2020 to identify all MC twins with concordant and discordant CHD with outcome of a livebirth. Dichorionic and conjoined twins were excluded from this study. Institutional Review Board (IRB) was obtained from UCLA (IRB #17–000925). Patient medical record numbers were used to obtain demographic information and were linked to the neonatal medical record numbers. Additional variables related to known risk factors for CHD independent of chorionicity, such as advanced maternal age, family history of CHD, high maternal pre-pregnancy body mass index (BMI), diabetes, and conception with assisted reproductive technology (ART) were also abstracted [14, 15]. All chart-abstracted information was stored in a de-identified research database. Chorionicity was confirmed by both an early perinatal ultrasound and by review of final placental pathology after delivery. CHD diagnoses were determined by postnatal echocardiography along with surgical operative reports, if available.

Concordant CHD was defined as a twin pair with an identical CHD diagnosis. Discordant CHD was defined as twin pairs with one affected and one unaffected fetus or twin pairs with different CHD diagnoses. Chart review was conducted to collect genetic test data, CHD diagnoses, extra-cardiac anomalies, and invasive (clinical) genetic testing results were obtained from prenatal amniocentesis samples or postnatal serum samples. Specifically, genetic test modalities included in analysis were karyotypes, microarrays, and fluorescence in situ hybridization (FISH) collected as an amniocentesis and/or chorionic villus sampling (CVS). Genetic screening, such as non-invasive prenatal screening and state analyte screening were not included.

Categories of CHD diagnoses were assigned based on the International Nomenclature for Congenital Heart Surgery (INCHS) by two providers specialized in fetal echocardiography and pediatric cardiology [16]. CHD were classified as one of the following: septal defects, pulmonary venous anomalies, systemic venous anomalies, right heart lesions, left heart lesions, single ventricle, transposition of the great arteries, and thoracic arteries or veins, which includes aortic arch, coronary artery, and ductus arteriosus anomalies.

Each diagnosis was further categorized by severity, with Category 1 indicating low risk of hemodynamic instability in the delivery room, Category 2 minimal risk of hemodynamic instability but requiring postnatal surgical intervention, Category 3 with likely hemodynamic instability requiring immediate specialty care, and Category 4 with expected hemodynamic instability requiring immediate surgical intervention.

Descriptive analysis including frequencies, means, medians, standard deviations, and interquartile range were conducted for all maternal variables, CHD diagnoses, and neonatal variables. Continuous variables were analyzed using the Student t-test and categorical variables were analyzed using the Fisher Exact test.

## Results

**Fig 1:**
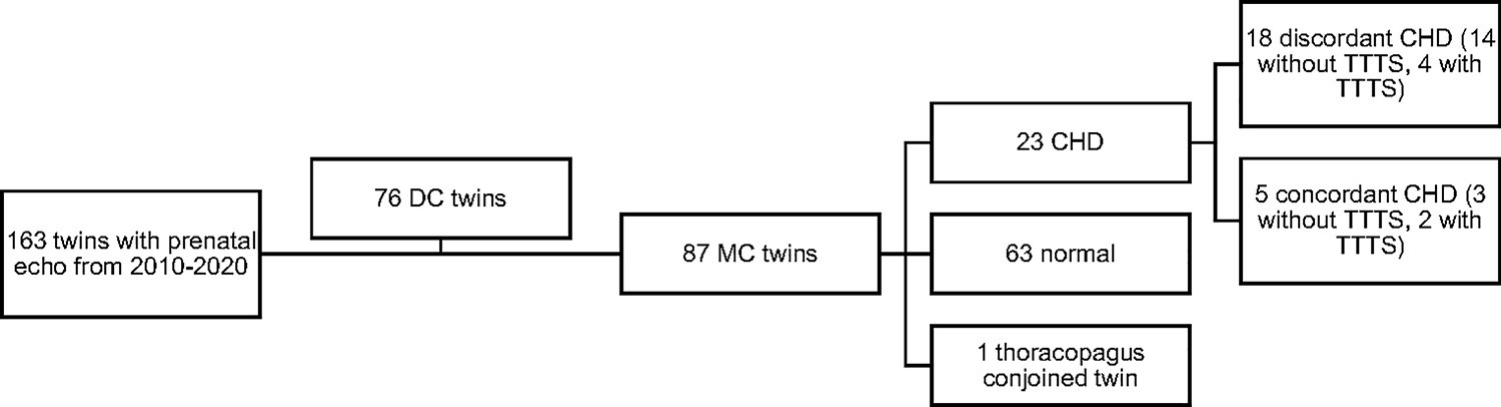
Study population of twins evaluated. DC, dichorionic; MC, monochorionic; CHD, congenital heart defects; TTTS, twin-to-twin transfusion syndrome.

Of the 163 twin pregnancies identified, 87 were MC twins (53.3%). Twenty-three MC twin pairs (46 neonates) had CHD (n = 5 concordant CHD, n = 18 discordant CHD pairs) (Fig 1). Table 1 displays the spectrum of CHD in discordant and concordant groups for this population. The most common lesions were septal defects (56% and 45.5% in concordant and discordant cohort) and right heart lesions (40% and 18.2% in concordant and discordant cohort). Lesions present in only the discordant pairs were systemic venous anomalies (4.5%), left heart lesions (9.1%), and thoracic arteries and veins (13.6%). There was no difference in the spectrum or severity of CHD between the two groups (p>0.05).

**Table 1:** Spectrum of CHD in concordant and discordant MC twins, as diagnosed by prenatal echocardiography.

Diagnosis^1^^,^^2^	Concordant CHD Cohort (n = 10)^3^	Discordant CHD Cohort (n = 22)^3^
Septal defects	6 (60%)	10 (45.5%)
Systemic venous anomalies	0 (0%)	1 (4.5%)
Right heart lesions	4 (40%)	4 (18.2%)
Left heart lesions	0 (0%)	2 (9.1%)
Transposition of the great arteries	0 (0%)	2 (9.1%)
Thoracic arteries/veins	0 (0%)	3 (13.6%)
CHD severity category^4^	1 (0)	1 (0)^5^

Data are no. (%) or median (IQR).

^1^Based on the International Nomenclature for Congenital Heart Surgery.

^2^p = 0.53.

^3^Includes only neonates affected by CHD.

^4^Category 1 = low risk of hemodynamic instability in the delivery room, Category 2 = minimal risk of hemodynamic stability but requiring postnatal surgical intervention, Category 3 = likely hemodynamic instability requiring immediate specialty care, Category 4 = expected hemodynamic instability requiring immediate surgical intervention.

^5^p = 1.

There was no difference in maternal demographic and risk factors in the two groups based on maternal co-morbidities or known risk factors for CHD, including pre-pregnancy BMI, diabetes, hypertensive disorders of pregnancy, and others (p>0.05) (Table 2). Antepartum length of stay (LOS) on labor and delivery was notably longer for the mothers in the discordant group than those in the concordant group, 10.61 versus 5.20 days, but this difference was not significant (p = 0.101). Five (21.7%) in-vitro fertilization (IVF) pregnancies were identified in this population, all of which were diagnosed with discordant heart lesions. Family history of CHD was rare in both cohorts, with none identified in the concordant and 11.1% in the discordant twin pair group.

**Table 2:** Maternal demographics in concordant and discordant MC twins (p = 0.634).

Variable	Concordant CHD cohort (N = 5)	Discordant CHD cohort (N = 18)	P-value
Maternal age (years)	30.40 ± 3.71	32.39 ± 7.51	0.426
Race			0.745
American Indian or Alaska Native	0 (0%)	0 (0%)	
Asian	1 (20%)	1 (5.6%)	
Black or African American	0 (0%)	0 (0%)	
Native Hawaiian or Other Pacific Islander	0 (0%)	0 (0%)	
White	4 (80%)	14 (77.8%)	
Other	0 (0%)	2 (11.1%)	
Denied	0 (0%)	1 (5.6%)	
Ethnicity			1.00
Hispanic or Latino	1(20%)	4 (22.2%)	
Not Hispanic or Latino	4 (80%)	13 (72.2%)	
Other	0 (0%)	1 (5.6%)	
Pre-gravid BMI (kg/m^2^)	25.70 ± 5.62	24.75 ± 6.26	0.754
Pre-gestational Diabetes Mellitus	0 (0%)	0 (0%)	
Gestational Diabetes	0 (0%)	4 (22.2%)	0.539
Chronic Hypertension	0 (0%)	1 (5.6%)	1.00
Hypertensive Disorders of Pregnancy	2 (40%)	4 (22.2%)	0.576
Urinary tract infection	1 (20%)	0 (0%)	0.217
Family History of CHD	0 (0%)	2 (11.1%)	1.00
IVF pregnancy	0 (0%)	5 (27.8%)	0.545
Maternal length of stay (L&D)	5.20 ± 1.30	10.61 ± 13.05	0.101
First trimester exposure to SSRI	1 (20%)	1 (5.6%)	0.395
Illicit drug use in pregnancy	0 (0%)	0 (0%)	
Diagnostic genetic testing	4 (80%)	9 (50%)	0.339
Abnormal genetic results	1 (20%)	1 (5.6%)	0.395

BMI, body mass index; IVF, in-vitro fertilization; SSRI, selective serotonin reuptake inhibitor.

Data are mean ± s.d. or no. (%).

Diagnostic genetic testing results were available for 80% of the concordant and 50% of the discordant cohort. There was one abnormal result in each group (20% concordant, 5.6% discordant), with no difference in rate of abnormal genetic results between the two groups (p = 0.395).

Details of the CHD diagnoses, neonatal clinical, genetic, and outcome data with associated extra-cardiac malformations for this population are listed in Tables 3–5. CHD severity was similar in both cohorts, with a median CHD severity category of 1 for both concordant and discordant twins. In the concordant cohort, one twin pair with right heart lesions had a *NOTCH1* variant c.5720C>T and thumb abnormalities identified for both neonates. In the discordant cohort, renal anomalies were identified in three neonates, one twin pair with a VSD/normal phenotype, and one fetus with a PDA and PFO. One neonate with complete atrioventricular canal and pulmonary atresia had intestinal malrotation and heterotaxy syndrome with asplenia, consistent with right atrial isomerism. Overall, rates of malformations in our discordant cohort were low. None of the patients with malformations and CHD had abnormal family history or genetic testing. Placental malperfusion and/or vascular malformations were common in our study, with 4/5 (80%) of concordant and 9/18 (50%) of discordant pregnancies with either cord or placental findings.

**Table 3:** Clinical details and outcome data with associated extra-cardiac malformations for monochorionic twins concordant for CHD (excluding those with TTTS).

Chorionicity/amnionicity	Gestation age at birth	Birth weight (g)	Mode of Delivery	APGARs (1 min, 5min)	CHD Category	INCHS detail^16^	INCHS category^16^	Extra-cardiac malformations	Number of surgeries in 1^st^ year	Prenatal Genetics*	Postnatal Genetics	Placental Pathology	Outcome
Mono/di	35w1d	2620	Cesarean	9,9	1	VSD, multiple	Septal defect	None	0	Normal karyotype, microarray	None	Foci intervillous thrombohematoma <10% volume	Live birth
		3325	Cesarean	8,9	1	VSD, single	Septal defect	None	0	Normal karyotype, microarray	None	Foci intervillous thrombohematoma <10% volume	Live birth
Mono/di	33w3d	1638	Vag-Spont	8,8	2	Pulmonary atresia, VSD	Right heart lesion	Hypoplastic first metacarpal and proximal phalanx of right thumb	2	Normal karyotype, microarray, FISH 22q11.2	Exome sequencing heterozygous NOTCH1 c.5720C>T variant, maternal allele	None	Live birth
Mono/di	33w3	1742	Vag-Spont	8,9	2	TOF	Right heart lesion	Bifid thumb	2	Normal karyotype, microarray, FISH 22q11.2	Exome sequencing heterozygous NOTCH1 c.5720C>T variant, maternal allele	None	Live birth
Mono/di	32w6d	1700	Cesarean	8,9	1	Tricuspid disease, non Ebstein’s; Mitral regurgitation	Right heart lesion	Right inguinal hernia	0	None	None	None	Live birth
Mono/di	32w6d	1850	Cesarean	8,9	1	Tricuspid disease, non Ebstein’s	Right heart lesion	None	0	None	None	Hypercoiled cord, mild chronic deciduitis	Live birth

*prenatal genetics = diagnostic testing (amniocentesis or chorionic villus sampling).

**Table 4:** Clinical details and outcome data with associated extra-cardiac malformations for monochorionic twins discordant for CHD (excluding those with TTTS).

Chorionicity/amnionicity	Gestation age at birth	Birth weight (g)	Mode of Delivery	APGARs (1 min/5min)	CHD Category	INCHS detail^16^	INCHS category^16^	Extra-cardiac malformations	Number of surgeries in 1^st^ year	Prenatal Genetics*	Postnatal Genetics	Placental Pathology	Outcome
Mono/mono	28w3d	820	Cesarean	1,6	1	VSD, multiple	Septal defect	Duplicated left renal collecting system, mild hydronephrosis	0	Normal karyotype, FISH (13, 18, 21)	Normal karyotype, microarray	None	Live birth
		1160	Cesarean	8,8	N/A	N/A	N/A	Mild L hydronephrosis	0	Normal karyotype, FISH (13, 18, 21)	Normal karyotype, microarray	None	Live birth
Mono/di	31w0d	1385	Vag-Spont	6,8	2	Aortic stenosis, valvar; Mitral stenosis, supravalvar mitral ring	Left heart lesion	None	2	Normal karyotype, microarray	Normal karyotype, microarray	420 g, <10%le weight for GA	Live birth
		1740	Vag-Spont	6,8	N/A	N/A	N/A	None	0	Normal karyotype, microarray	None	420 g, <10%le weight for GA	Live birth
Mono/di	32w2d	1595	Cesarean	8,9	N/A	N/A	N/A	None	0	Normal karyotype	Microarray 1.5Mb copy loss involving 15q13.2q-13.3 and two copy gains on 7q31.31 and Xp22.2	Hypocoiled cord	Live birth
		850	Cesarean	7,8	1	ASD, secundum	Septal defect	Left inguinal hernia	0	Normal karyotype	Microarray 1.5Mb copy loss involving 15q13.2q-13.3 and two copy gains on 7q31.31 and Xp22.2	Hypocoiled cord	Live birth
Mono/di	32w6d	2230	Cesarean	9,9	1	ASD, secundum; pulmonary artery stenosis, branch central	Septal defect	None	0	None	None	None	Live birth
		2120	Cesarean	9,9	N/A	N/A	N/A	None	0	None	None	None	Live birth
Mono/di	34w0d	1330	Cesarean	9,10	N/A	N/A	N/A	None	0	Microarray uniparental disomy 2 but PCR of C2 normal biparental inheritance	None	75% vascular distribution	Live birth
		2065	Cesarean	8,9	1	Tricuspid valve disease, non Ebstein’s related	Right heart lesion	None	0	Microarray uniparental disomy 2 but PCR of C2 normal biparental inheritance	None	25% vascular distribution, hypercoiled, velamentous insertion, infarction and intervillous thrombohematoma	Live birth
Mono/di	34w1d	1970	Cesarean	9,9	N/A	N/A	N/A	None	0	None	None	Bivascular cord	Live birth
		1760	Cesarean	6,8	1	VSD, single	Septal defect	None	0	None	None	Normal	Live birth
Mono/di	34w6d	2300	Cesarean	8,7	1	VSD, single	Septal defect	None	0	None	None	None	Live birth
		2315	Cesarean	8,9	N/A	N/A	N/A	None	0	None	None	None	Live birth
Mono/di	35w4d	2380	Cesarean	9,9	N/A	N/A	N/A	None	0	None	None	None	Live birth
		2350	Cesarean	9,9	1	Tricuspid valve disease, non Ebstein’s related	Right heart lesion	None	0	None	None	Hypocoiled cord	Live birth
Mono/di	36w4d	2375	Cesarean	8,8	3	DORV, VSD type	Transposition of the great arteries	None	5	Unknown	Normal karyotype, microarray, exome sequencing	Size >90%ile	Death at 3 months of age
		2680	Cesarean	9,9	N/A	N/A	N/A	None	0	Unknown	None	Size >90%ile	Live birth
Mono/di	36w5d	2220	Cesarean	8,8	2	AVC, complete; pulmonary atresia	Septal defect	Intestinal malrotation, hydronephrosis, heterotaxy syndrome with asplenia	6	Normal karyotype, microarray	Normal karyotype, microarray, exome sequencing	None	Live birth
		2260	Cesarean	8,9	N/A	N/A	N/A	None	0	Normal karyotype, microarray	None	None	Live birth
Mono/di	37w4d	2610	Vag-Spont	1,1	1	Systemic venous anomalies	Thoracic arteries and veins	None	0	None	None	Single UA, marginal insertion, 30% chorionic plate	Live birth
		3660	Vag-Spont	8,9	1	Tricuspid valve disease, non Ebstein’s related	Right heart lesion	None	0	None	None	70% chorionic plate	Live birth
Mono/di	38w2d	2380	Cesarean	9,9	N/A	N/A	N/A	None	0	None	None	None	Live birth
		2995	Cesarean	9,9	1	VSD, multiple	Septal defect	None	0	None	None	None	Live birth
Mono/mono	34w0d	1990	Cesarean	8,9	1	AVC, complete	Septal defect	None	0	None	Normal karyotype, microarray	Single UA	Live birth
		1930	Cesarean	8,9	2	DORV, VSD type	Transposition of the great arteries	None	2	None	Normal karyotype, microarray	None	Live birth
Mono/di	35w4d	2167	Cesarean	9,9	2	HLHS	Left heart lesion	None	2	None	None	None	Live birth
		2000	Cesarean	9,9	N/A	N/A	N/A	None	0	None	None	None	Live birth

*prenatal genetics = diagnostic testing (amniocentesis or chorionic villus sampling).

**Table 5:** Clinical details and outcome data with associated extra-cardiac malformations for monochorionic twins with TTTS.

Chorionicity/amnionicity	TTTS Stage	Gestation age at birth	Birth weight (g)	Donor or Recipient	Treatment	EGA at treatment	Mode of Delivery	APGARs (1 min/5min)	CHD type	INCHS detail^16^	INCHS category^16^	Extra-cardiac malformations	Number of surgeries in 1^st^ year	Prenatal Genetics*	Postnatal Genetics	Placental Pathology	Outcome
Mono/di	Stage 2	34w0d	1983	Recipient	Cerclage and laser ablation	133	Cesarean	9,9	1	ASD, secundum	Septal defect	None	0	Normal karyotype, microarray	None	Lymphoplasmacytic chronic deciduitis—Intervillous fibrin deposition with villous infarction	Live birth
			2105	Donor			Cesarean	5,7	1	ASD, secundum	Septal defect	None	0	Normal karyotype, microarray	None		Live birth
Mono/di	Stage 1	33w4d	1721	Donor	Amnio-reduction	174	Cesarean	7,8	1	ASD, sinus venosus	Septal defects	None	0	Normal karyotype	None	Velamentous insertion, hypercoiled cord, multifocal accelerated villous maturation, chorangioma 0.8 cm, >97%ile size	Live birth
			1393	Recipient			Cesarean	9,9	1	ASD, secundum	Septal defects	None	0	Normal karyotype	None	>97%ile size	Live birth
Mono/di	Stage 1	24w4d	560	Recipient	None	N/A	Vag-Spont	5,7	1	Patent ductus arteriosus	Thoracic arteries and veins	Parietal porencephalic cysts	1	None	None	Vasa previa	Death at 7 weeks of age
			570	Donor			Vag-Spont	6,8	1	Secundum, ASD	Septal defects	None	1	None	None	None	Live birth
Mono/di	Stage 2	34w1d	1785	Recipient	2 Laser ablations	128, 135	Cesarean	9,9	1	Persistent left superior vena cava (PLSV)	Systemic venous anomaly	None	0	Normal karyotype, microarray	None	Unknown	Live birth
			2035	Donor			Cesarean	8,9	1	Tricuspid valve disease, non Ebstein’s	Right heart lesion	None	0	Normal karyotype, microarray	None	Unknown	Live birth
Mono/di	Stage 4	35w4d	2215	Donor	None	N/A	Cesarean	8,8	N/A	N/A	N/A	None	0	None	None	None	Live birth
			3010	Recipient			Cesarean	7,7	1	ASD, secundum	Septal defect	None	0	None	None	None	Live birth
Mono/di	Stage 1	31w0d	1285	Donor	Laser ablation	173	Cesarean	1,7	N/A	N/A	N/A	None	0	Normal karyotype, microarray	None	None	Live birth
			1460	Recipient			Cesarean	1,7	1	Patent ductus arteriosus, PFO	Thoracic arteries and veins	Bilateral hydronephrosis, mild right renal stenosis	0	Normal karyotype, microarray	None	None	Live birth

*prenatal genetics = diagnostic testing (amniocentesis or chorionic villus sampling).

Six (25%) twin pairs in the population were complicated by TTTS: 2 concordant (septal defects) and 4 discordant (thoracic arteries and veins 25%, septal defect 25%, systemic venous anomaly 13%, and right heart lesion 13%) (Table 5). All recipient twins were diagnosed with a heart lesion and 4/6 (67%) donor twins were also affected by CHD. There were no abnormal genetic test results in the twin pairs complicated by TTTS.

Neonatal outcome was analyzed based on concordance of CHD (Table 6). The average gestational age of delivery in both cohorts was ~33 weeks of gestation, with birthweight of 2007g in the concordant and 1960g in the discordant group. The majority of patients, 80% in the concordant cohort and 83% in the discordant cohort, were delivered via cesarean. Almost all neonates were hospitalized in the neonatal intensive care unit (NICU), with a median LOS of 32 (22.75) days in the concordant and 22 (47) days in the discordant cohorts. There was no difference in any neonatal outcomes studied between the two groups (p>0.05) (Table 6). When neonatal outcomes were analyzed to only include neonates affected by CHD in concordant (n = 10) and discordant (n = 22) pairs (Table 7), there was no difference in outcomes, though there was a greater gap between the number of surgeries in the discordant (0.86 ± 1.67) compared to the concordant group (0.40 ± 0.84 surgeries) (Table 7).

**Table 6:** Neonatal outcomes and clinical details in concordant and discordant CHD groups.

Variable	Concordant CHD cohort (N = 10)	Discordant CHD cohort (N = 36)	P-value
Sex			0.725
Female	4 (40.00%)	18 (50%)	
Male	6 (60.00%)	18 (50%)	
Gestational age at birth	33w5d ± 5d	33w5d ± 3d	0.919
Birthweight (g)	2007.70 ± 568.34	1959.92 ± 677.32	0.825
Mode of Delivery			1.00
Vaginal	2 (20.00%)	6 (16.7%)	
Cesarean delivery	8 (80.00%)	30 (83.3%)	
APGAR 1 minute	7.90 ± 1.20	7.20 ± 2.46	0.215
APGAR 5 minute	8.60 ± 0.70	8.20 ± 1.51	0.234
NICU Admission	10 (100.0%)	31 (86.1%)	0.570
NICU LOS	32 (22.75)	22 (47)	0.827
Number of surgeries in first year	0.40 ± 0.84	0.53 ± 1.36	0.718
Outcome			1.00
Live Birth	10 (100%)	34 (94.4%)	
Neonatal Death (<28 days)	0 (0%)	0 (0%)	
Infant Death (>28 days)	0 (0%)	2 (5.6%)	

NICU = neonatal intensive care unit, LOS = length of stay, Data are mean ± s.d., no. (%), or median (IQR).

**Table 7:** Neonatal outcomes and clinical details in concordant and discordant CHD groups for affected neonates only.

Variable	Concordant CHD cohort (N = 10)	Discordant CHD cohort (N = 22)	P-value
Sex			0.711
Female	4 (40.00%)	11 (50%)	
Male	6 (60.00%)	11 (50%)	
Gestational age at birth	33w5d ± 5d	33w4d ± 3w5d	0.740
Birthweight (g)	2007.70 ± 568.34	1960.32 ± 792.91	0.850
Mode of Delivery			1.00
Vaginal	2 (20.00%)	5 (22.7%)	
Cesarean delivery	8 (80.00%)	17 (77.3%)	
APGAR 1 minute	7.90 ± 1.20	6.77 ± 2.60	0.103
APGAR 5 minute	8.60 ± 0.70	7.86 ± 1.78	0.104
NICU Admission	10 (100%)	19 (86.4%)	0.534
NICU LOS	32 (22.75)	23.5 (49.75)	0.471
Number of surgeries in first year	0.40 ± 0.84	0.86 ± 1.67	0.305
Outcome			1.00
Live Birth	10 (100%)	20 (90.9%)	
Neonatal Death (<28 days)	0 (0%)	0 (0%)	
Infant Death (>28 days)	0 (0%)	2 (9.1%)	

NICU = neonatal intensive care unit, LOS = length of stay, Data are mean ± s.d., no. (%), or median (IQR).

## Discussion

We compared a modern cohort of MC twins discordant and concordant for CHD to describe the spectrum of heart lesions identified in these two groups and to detail maternal demographic and pregnancy outcomes. The spectrum of CHD described in this study includes septal defects, systemic venous anomalies, right heart lesions, left heart lesions, transposition of the great arteries (TGA), and thoracic arteries/veins, with the most common lesions in both concordant and discordant cohorts being septal defects (60% and 45.5% in concordant and discordant cohort) and right heart lesions (40% and 18.2% in concordant and discordant cohort).

There was similar severity in presentation of CHD, as evidenced by equivalent median CHD severity category scores of affected fetuses in the concordant and discordant pregnancies. AlRais et al. described the spectrum of CHD in a discordant monochorionic cohort of 29 pregnancies, with 10% septal defects and 14% right heart lesions, comparable to our findings. In their cohort, all discordant twin pairs had one affected and one unaffected twin [5]. The majority of our non-TTTS discordant cohort (12/14, 86%) had one normal twin, though in the TTTS discordant subgroup, 50% of pregnancies resulted in both twins with CHD of differing type and severity. These results are consistent with existing data demonstrating that septal defects, are more common in MC twins than in singletons and abnormal blood flow due to TTTS preferentially affects cardiovascular function of the right ventricle in MC twins with associated development of right heart anomalies such as pulmonary atresia [17]. Defects known to be more significantly influenced by genetics, such as Tetralogy of Fallot (TOF), are reported to be equally prevalent in MC twins and singletons [1, 2, 18].

Although somatic mosaicism and somatic chromosomal abnormalities have been described in MC twins, we found no evidence for genetic discordance in twin pairs with discordant CHD [19]. This fact, coupled with the higher rates of discordant (78.2%) versus concordant (21.7%) CHD, low incidence of family history of CHD, and low incidence of genetic abnormalities (8.7%) in this population, suggests that genetic differences in coding regions of the genome are non-contributary in many cases of discordant CHD. There was one abnormal test result in our discordant cohort, a twin pair with a septal defect and left inguinal hernia in one twin and normal finding in the other twin. Their microarray had a 1.5 Mb copy loss of 15q13.2q-13.3 and two copy gains on 7q31.31 and Xp22.2. The copy gains are of unknown significance. The copy loss on chromosome 15 has been described in patients with neurodevelopmental defects but not cardiac abnormalities [20]. The yield of genetic testing in detecting CHD-specific mutations in discordant twins, especially those affected by TTTS, is low, as demonstrated by our negative findings and by studies of copy number variant and exosome sequencing analysis in discordant MC twins [4, 21]. On the contrary, genetic tests with high resolution can be diagnostic in concordant pairs, as evidenced by the *NOTCH1* gene variant associated with non-syndromic TOF that was seen in the twin pairs with right heart lesion and thumb malformations [22]. The utility of in-depth genetic analysis has also been demonstrated in singletons, especially in patients with complex and syndromic CHD [23].

We provide hypothesis generating data that discordant CHD pairs may benefit from tests that provide insight into gene regulation. Although our study did not analyze epigenetic influences, it is important to note that certain post-translational modifications may be significant in the mechanism of discordant CHD development in MC twins. For example, epigenetic analyses found differentially methylated regions in promoters for genes involved in cardiac development in discordant twins [3]. Abu-Halima et al. recently found that microRNAs involved in molecular transport and adrenergic signaling in cardiomyocytes are enriched in CHD compared to non-CHD twins [23].

The etiological question remains how monochorionic twins, presumed to have an identical genotype, can display discordant phenotypes for congenital malformations such as CHD. Our data supports the conclusion that environmental influences likely play a significant role in the pathophysiology of discordant CHD. Factors affecting normal growth and development leading to CHD are hypothesized to occur as early as the first weeks of life, supported by high rates of cardiac looping and laterality defects in discordant CHD as well as the higher prevalence of CHD in twins conceived by assisted reproductive technology [5, 15]. In the discordant cohort, 27.8% had undergone the process of IVF compared to 0% of our concordant cohort, highlighting the influence of gamete manipulation, culture, and embryo implantation on non-familial CHD. In later gestation, although MC twins share a placenta, they do not necessarily experience identical growth environments. Anastomoses between fetal circulations play a significant role in intrauterine development and have the potential to cause syndromes such as TTTS [24]. In this population, the high frequency of TTTS (26%), with incidence of discordance twice that of concordance, indicates the critical role of hemodynamics in the pathophysiology of discordant CHD. The growth conditions for the recipient twin are significantly different than that of the donor twin, subsequently influencing cardiac development. A study analyzing ventricular strain changes in MC twins affected by TTTS shows that at all Quintero stages, recipient left ventricular strain was reduced compared with donors [25]. In stages 3 and 4, recipient right ventricular strain was reduced compared with donors [25]. The relative risk of right ventricular outflow tract obstruction is 70 times that of singletons for MC twins with TTTS, emphasizing the likely importance of hemodynamics on the development of cardiac structures in the second trimester, even after the heart has fully formed [1].

The strengths of this study include a large sample size of discordant twins with a novel comparison to a concordant twin cohort to elucidate environmental risk factors for CHD based on multiple maternal factors and neonatal outcomes. Our study includes a modern cohort with diagnostic testing results achieved using current genetic technology. We were able to follow neonates until at least one year of life, given that all were cared for by a single pediatric cardiology center. Although these results enhance our understanding of the possible mechanism for the development of discordant CHD, it is not possible to establish a temporal relationship between clinical influences and disease development. There are several limitations in our study, including use of a cohort from one specialized center, making this sample subject to selection bias. For example, the prevalence of CHD in our cohort of MC twins is 26.4% compared to literature reporting that about 5.9% of MC twins receive a diagnosis of CHD [1]. The analysis of this specialized cohort, however, also strengthens our findings by allowing for complete data collection with excellent follow up. Additionally, this study details genetic analysis for 80% of the concordant and 50% of the discordant cohort but lacks genetic test results for the remainder of the twin pairs, limiting our ability to draw statistically significant comparisons between the two cohorts with regards to contributing genetic abnormalities. We used abnormal prenatal echocardiography as the criteria for enrollment, and given the low sensitivity of this modality especially in obese patients, CHD diagnosed only postnatally would not be included in our study. Although prenatal fetal echocardiography is effective in early identification of major congenital heart defects, some heart defects with the potential to evolve *in utero* may be missed on prenatal echocardiography [26]. Therefore, this study errs on the side of capturing more severe CHD or CHD more easily detected on prenatal ultrasound. Future studies analyzing both prenatal and postnatal echocardiography records of twins who did not receive a prenatal diagnosis may be helpful in obtaining a representative population of MC twins with discordant CHD.

Our findings emphasize the importance of evaluating MC twins for CHD, especially those conceived through assisted reproductive technology or affected by TTTS. Increasing availability of genetic tests that are sensitive to discrepancies in epigenetic programming are needed in discordant CHD, given the low yield of traditional sequencing analyses for detecting cardiac specific alterations. Diagnostic evaluation that can detect early changes in twin cardiac hemodynamics that are known risk factors for discordant CHD may also improve prenatal detection of cardiac malformations that have implications for neonatal morbidity and mortality. We describe genetic evaluation and fetal and maternal clinical variables in a modern cohort of MC twins to demonstrate the significance of environmental influences and hemodynamics on the development of discordant CHD, elucidating target areas for early detection and risk modification.
